# Optical Coherence Tomography Findings in Nodular Anterior Scleritis due to Post-Streptococcal Syndrome

**DOI:** 10.18502/jovr.v16i1.8260

**Published:** 2021-01-20

**Authors:** Kais BenAbderrahim

**Affiliations:** ^1^Department of Ophthalmology, University Hospital of Médenine, Faculty of Medicine, Sfax Univer-sity, Tunisia

**Keywords:** Scleritis, Optical Coherence Tomography, Poststreptococcal Syndrome

## Abstract

**Purpose:**

To report a case of nodular anterior scleritis due to poststreptococcal syndrome using optical coherence tomography imaging.

**Case Report:**

A 41-year-old woman with a history of acute rheumatic fever presented with a nodular anterior scleritis. Common causes were excluded. Optical coherence tomography of sclera showed enlarged vessels, inflammatory infiltrates, separated fibers, and a serous detachment. Laboratory investigations showed an elevated erythrocyte sedimentation rate, raised anti-streptolysin O titer, and the presence of group A streptococcus in the throat. The scleritis rapidly improved with penicillin treatment.

**Conclusion:**

Poststreptococcal syndrome should be considered in the etiology of non-necrotizing anterior scleritis.

##  INTRODUCTION

Poststreptococcal syndrome (PSS) includes all nonsuppurative complications of infections with group A streptococci.^[1,2]^ It appears as an immune-mediated reaction in any tissue of the body.^[1]^ Acute rheumatic fever and acute glomerulonephritis are the common entities of PSS which most frequently involves young patients.^[1,2]^ Ocular involvement of PSS is uncommon and rare.^[1,2]^ More precisely, PSS is not considered among the common causes of scleritis.^[3,4]^ Scleritis is an inflammatory condition characterized by ocular pain and redness of the sclera.^[3]^ It can threaten vision in severe cases.^[3]^ Previous studies reported that optical coherence tomography (OCT) showed different changes in the sclera within each grade of active scleritis.^[5,6]^ Our aim is to describe a rare case of nodular anterior scleritis due to PSS using OCT imaging.

##  CASE REPORT

**Figure 1:**
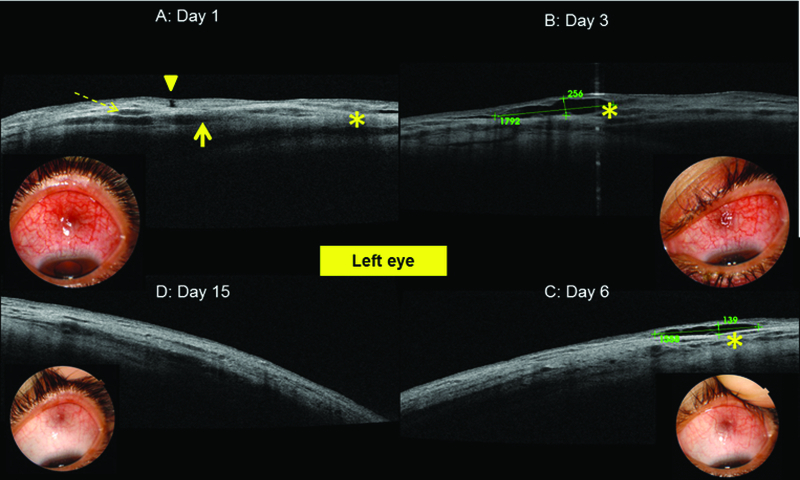
(A) Optical coherence tomography images of the left eye showed separated fibers (dashed arrow), enlarged vessels, inflammatory infiltrates (solid arrow), serous detachment between them (asterisk), and the site of conjunctival biopsy (arrow head). (B, C, D) Evolution after penicillin treatment.

**Figure 2:**
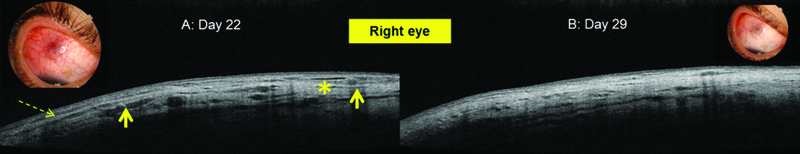
(A) Optical coherence tomography showed separated fibers (dashed arrow) and inflammatory infiltrates (solid arrow) due to recurrence of anterior scleritis in the right eye. (B) Resolution of signs in the final examination.

A 41-year-old woman had a history of an acute rheumatic fever (ARF) treated with penicillin in childhood. She presented with a two-day history of redness and pain in her left eye. Symptoms occurred few days after an acute pharyngitis. She reported a history of a similar episode in her right eye a year ago. Best-corrected visual acuity was 20/20 in both eyes. Slit lamp examination revealed a nodule in the superior sclera with hyperemia and chemosis around it [Figure 1]. Ocular examination and funduscopy excluded all forms of uveitis or suppurative infections. B-scan ultrasonography showed no abnormalities in the posterior segment of the left eye. Spectral Domain OCT (3D OCT-1Maestero; Topocon, Japan) of the sclera showed enlarged vessels, inflammatory infiltrates (hyporeflective spaces), separated fibers, and a serous detachment between them [Figure 1]. The scleral thickness at the level of visible layers was 659 µm on the nodular area and 555 µm around it. A surgical punch biopsy of conjunctiva and Tenon's tissue was performed at admission. Histopathologic exam revealed mild and nonspecific inflammation and excluded bacterial or parasitological infections. Investigations showed negative results for tuberculosis, syphilis, and rheumatoid arthritis (chest X-ray, throat culture, tuberculin skin testing, syphilis serology, antinuclear antibodies, rheumatoid factor). Laboratory tests showed high erythrocyte sedimentation rate (35 mm in the first hour), raised anti-streptolysin O (ASO) titer of 545 units/ml, the presence of group A *Streptococcus* in the throat culture, C-reactive protein of 1 mg/l, a white blood count of 5600/mm${}^{3}$ (lymphocytes: 51%), and normal levels of blood electrolytes, glycemia, and azotemia. The patient received benzathine benzylpenicillin (extencilline: 1.2 million units intramuscularly twice monthly for three months). Examination showed rapid improvement within six days and remarkable resolution of signs after two weeks [Figure 1]. OCT demonstrated accumulation of the liquid in the sub-Tenon's space after 6 days and improvement of the fibrous structure of the sclera after 15 days. Recurrence of signs was observed in the right eye after 22 days [Figure 1]. The patient declared that she had not received the second dose of extencilline. OCT revealed hyporeflective areas due to an inflammatory fluid in the right sclera [Figure 2] and normal findings in the left eye. Extencilline treatment was administrated with the same doses in addition to oral corticosteroids. Complete recovery of signs was noticed after one week [Figure 2]. Normal level of ASO titers was reached after two months.

##  DISCUSSION

In this report, we described an uncommon case of nodular anterior scleritis induced by presumed PSS. Findings supporting the diagnosis of PSS scleritis were the history of ARF, pharyngitis, biopsy results, the high erythrocyte sedimentation rate, the raised ASO antibody titer, the evidence of streptococcal infections, the rapid response to penicillin, the early recurrence when the patient stopped penicillin treatment, and the negative results for all main diseases responsible for scleritis.^[1,6,7,8]^

Reported cases of an ocular involvement of PSS include scleritis, in addition to uveitis, and rarely episcleritis, conjunctivitis, Brown's syndrome, optic disc edema, posterior scleritis, and glaucoma.^[2,7,8,9,10,11,12]^ However, previous studies reported PSS as an uncommon cause of uveitis or scleral inflammation.^[2]^ To our knowledge, PSS was not figured among the possible causes of nodular anterior scleritis.^[3,4]^ Anamnesis, clinical examination, and laboratory investigations were helpful to exclude causes of necrotizing forms of scleritis and to suggest PSS. Biopsy may be helpful in establishing the diagnosis in cases of scleritis, but cannot rule out special scleral diseases especially in cases of non-necrotizing scleritis.^[6]^

According to the anatomo-clinical classification of scleritis, there are two forms of scleritis: anterior and posterior.^[6]^ Anterior scleritis is divided into nodular, diffuse, and necrotizing scleritis. The nodular scleritis has two forms: necrotizing and non-necrotizing forms. OCT is useful to show the scleral changes and to classify the scleral inflammation. Moreover, this tool is helpful in distinguishing all forms of anterior scleritis and monitoring them.^[5,6]^ The common scleral changes in anterior scleritis were separated fibers, dilated vessels, hyporeflective spaces and high thickness of sclera.^[5,6]^ Although thickening of the episcleral layer was observed in both episcleritis and anterior scleritis, scleral layers were not affected in episcleritis.^[13]^ OCT imaging is useful for distinguishing between non-necrotizing and necrotizing anterior scleritis. In the cases of non-necrotizing scleritis, the collagen fibers were simply separated and associated with an extracellular fluid without necrosis of tissue. In the second form, scleral OCT showed destructive changes, in which any hyporeflectivity of the deep layers of the nodule corresponded to liquified tissues.^[2][5,6][10][13]^ In addition, this tool is useful to grade anterior scleritis from mild to severe when the activity signs reach the deeper sclera and the suprachoroidal space.^[5]^ In this case, OCT findings strongly suggested non-necrotizing form of nodular scleritis as described in the previous reports.^[1,6,7,8][13]^

Sometimes, OCT can suggest the etiology of anterior scleritis. Common associated diseases were rarely found in the cases of non-necrotizing noninfectious scleritis.^[6]^ However, OCT was helpful in suggesting the etiology in some cases of necrotizing anterior scleritis. This tool showed destructive changes that involved the cornea, limbus, and the adjacent sclera in the case of a systemic vasculitis. However, the adjacent sclera was normal in the case of an idiopathic and rheumatoid-associated sclero-keratitis. The characteristic changes in rheumatoid arthritis are those of a venular occlusive scleritis affecting the vessels of the episcleral plexus.^[5,6][10]^

In this patient, presumed PSS scleritis rapidly improved with just penicillin. Such a finding have been previously reported.^[8]^ Penicillin prophylaxis is needed to prevent PSS complications and recurrence.^[1,7,8,12]^ Presumed PSS scleritis may be among refractory cases to standard corticosteroid treatment and may require penicillin for treatment.^[7]^ The fluid space, that has been seen at admission (before the biopsy) and that has been increased at the follow-up examination of the left eye, seemed to be a new clinical event.

In conclusion, PSS should be considered in the etiology of nodular anterior scleritis. ASO titer should be performed in any patient suffering from anterior scleritis and having a history of streptococcal infections. OCT may be helpful in the diagnosis and follow-up of scleral lesions.

##  Financial Support and Sponsorship

Nil.

##  Conflicts of Interest

There are no conflicts of interest.
